# Efficacy and safety of biosimilar insulins compared to their reference products: A systematic review

**DOI:** 10.1371/journal.pone.0195012

**Published:** 2018-04-18

**Authors:** Carolyn Tieu, Eleanor J. Lucas, Mindi DePaola, Lori Rosman, G. Caleb Alexander

**Affiliations:** 1 Department of Epidemiology, Johns Hopkins Bloomberg School of Public Health, Baltimore, MD United States of America; 2 Center for Drug Safety and Effectiveness, Johns Hopkins University, Baltimore, MD United States of America; 3 Pharmerit International, Bethesda, MA United States of America; 4 Welch Medical Library, School of Medicine, Johns Hopkins University, Baltimore, MD; 5 Division of General Internal Medicine, Department of Medicine, Johns Hopkins Medicine, Baltimore, MD United States of America; University of Southampton Faculty of Medicine, UNITED KINGDOM

## Abstract

**Importance:**

For nearly a century, no generic form of insulin has been available in the United States. However, the first biosimilar insulin, Basaglar, was approved by the U.S. Food and Drug Administration in 2015, and subsequently Admelog and Lusduna in 2017.

**Objective:**

To summarize the scientific evidence comparing the safety, efficacy, pharmacokinetics, and pharmacodynamics of biosimilar and reference insulin products.

**Data sources:**

We conducted a systematic review using PubMed, Cochrane, Embase, Latin America and Caribbean Health Sciences, South Asian Database of Controlled Clinical Trials, and IndiaMED from their inception through January 14, 2018.

**Study selection:**

We included randomized controlled trials (RCTs) comparing safety, clinical efficacy, pharmacokinetics and pharmacodynamics of any biosimilar insulin with a reference product in adults regardless of sample size and location.

**Data extraction and synthesis:**

Two researchers independently reviewed all titles, abstracts and text; extracted data; and performed quality assessments.

**Main outcomes and measures:**

Efficacy, safety, pharmacokinetics, and pharmacodynamics of biosimilar and reference insulin products

**Results:**

Of 6945 articles screened, 11 studies were included in the data synthesis. LY2963016, Basalog, Basalin, and MK-1293 were compared to Lantus while SAR342434 was compared to Humalog. Three trials enrolled healthy volunteers, five enrolled type 1 diabetics, and two enrolled type 2 diabetics. One study enrolled both healthy and type 1 diabetics. Of the eleven studies, six examined pharmacokinetic and/or pharmacodynamic parameters and five examined clinical efficacy and immunogenicity. All studies included adverse events. All PK and/or PD studies showed that comparable parameters of biosimilar and reference products were within the pre-specified equivalence margins. Clinical studies suggested similar clinical efficacy and immunogenicity. Adverse events were similar between the groups across all studies.

**Conclusions and relevance:**

Few published studies have compared biosimilar and reference insulins, though those that did suggest that the biosimilars have comparable safety and clinical efficacy as its reference product.

## Introduction

Biopharmaceuticals, or products derived from living cells, represent a growing and important sector of the pharmaceutical marketplace [1]. They account for only a small proportion of all pharmaceutical treatments, yet are estimated to generate global revenues of $221 billion, making up about 20% of the pharmaceutical market, in 2017 [2]. While there are dozens of biopharmaceuticals currently available in the United States, one of the first to market was recombinant human insulin. Since its introduction in 1982, it has become the predominant means of producing insulin [3], and the global insulin market is estimated to exceed $43.6 billion by 2021 [4].

The first biosimilar insulin in United States, Basaglar, was approved for marketing in 2015, followed by Admelog and Lusduna in 2017 [5–7]. In addition, biosimilar insulins have been available in India (Glaritus, Glarvia, Basalog, Wosulin, Insugen, Biosulin), China (Basalin, Comonlin, Prandilin), Mexico (Bonglixan), Europe (Abasaglar) and other parts of Asia for more than a decade [8–11].

The growth of biosimilar insulins has generated considerable scientific and clinical interest, partly because in contrast to a typical small molecule product, insulin has well-defined primary, secondary and tertiary structures that are crucial for its biologic action [10]. Variations during manufacturing can have pronounced effects on insulin’s safety and efficacy [11–15]; for example, differences in its formulation can lead to changes in pharmacokinetics and pharmacodynamics [16–18]. Since manufacturing details are considered proprietary knowledge of the innovator, biosimilar manufacturers must develop their own production technologies [19].

Little is known regarding the comparability of biosimilar insulins and reference products across the world [19–21]. In a recent market research survey of patients with type 1 and type 2 diabetes, approximately 66% of respondents reported that they would switch to a hypothetical less expensive biosimilar insulin recommended by their provider [22], yet their most common concern was whether the biosimilar product would be as safe and effective as the reference product. While reviews of biosimilar insulins have been performed, many have focused on future markets and have been narrative in nature [21,23–26]. We conducted a systematic review to assess the scientific evidence comparing the efficacy, safety, pharmacokinetics, and pharmacodynamics of biosimilar and reference insulin products.

## Methods

### Data sources and searches

We conducted our review using PubMed, Cochrane CENTRAL, Embase, Latin America and Caribbean Health Sciences (LILACS), South Asian Database of Controlled Clinical Trials (SADCCT) and IndiaMED from their inception through January 14, 2018. We used a combination of controlled vocabulary and keywords to search for studies of biosimilar insulins. We did not include any date or language restrictions. All citations were imported into EndNote and duplicates were removed. We examined for the potential of publication bias by conducting a broad search of trial registries to examine for completed yet unpublished clinical trials. To do so, we searched ClinicalTrials.Gov, the WHO International Clinical Trials Registry Platform (ICTRP), and the EU Clinical Trials Register using keywords for biosimilars and biosimilar insulins (S1 Table).

### Study selection

We included randomized controlled trials (RCTs) comparing safety, clinical efficacy, pharmacokinetics or pharmacodynamics of any biosimilar insulin with a reference product in adults regardless of sample size and location. Trials were only selected if it had full text publications. We excluded studies that compared insulin without a biosimilar and where no English translation was available. Finally, we excluded studies that used an insulin pump since this may affect the clinical efficacy outcomes. References from studies chosen for inclusion were hand-searched to identify any additional relevant studies for analysis. Two researchers reviewed all titles, abstracts, and full text independently. All discordances between reviewers were resolved by consensus among the study team. A summary of search terms, databases, and inclusion criteria is presented in S1 Table.

### Data extraction and quality assessment

Two reviewers independently extracted data on the study design, study population, intervention and comparator, pharmacokinetic, pharmacodynamic, clinical efficacy, adverse events and immunogenicity. Pharmacokinetic parameters included area under the curve (AUC) and the drug maximum observed concentration (C_max_). Pharmacodynamic parameters included total glucose infusion during clamp procedures (G_total_) and maximum glucose infusion rate (R_max_). Clinical efficacy was defined on the basis of the primary outcomes of the trials, which was change in hemoglobin A1c (HbA1c) at different time points. Adverse events (AE) were defined as undesirable medical occurrences that may or may not have been casually related to the exposure in question and were extracted them as quantified in the included studies. Immunogenicity was extracted as a proportion of patients exposed to the biosimilar or reference product who developed antibodies to the product.

We used the Cochrane Risk of Bias Tool [27] to assess the risk of bias for randomized control trials. We assessed selection bias based on whether authors described the randomization sequence generation and allocation concealment. Performance and detection bias were based on whether the study was double blinded or whether the outcomes were influenced by knowledge of the allocated interventions. We assessed attrition bias based on how complete the data was for the primary end point and whether methods for addressing missing data were clearly described. We evaluated reporting bias on the basis of whether the study outcomes were pre-specified in the published study.

### Data synthesis and analysis

We grouped extracted data by study population and whether the study reported pharmacokinetic (PK), pharmacodynamics (PD), clinical efficacy (CE), adverse events (AE) and/or immunogenicity (IMM). For PK and PD outcomes, we assessed the parameters according to the specified equivalence margin and noted the geometric means ratio between the biosimilar and the reference product to demonstrate if comparability was achieved. For CE outcomes, we compared the hemoglobin A1c (HbA1c) and LS mean difference between the biosimilar and reference product. We analyzed AE by evaluating all patients with at least one AE, serious AEs, AEs requiring discontinuation of study, and deaths. For IMM, we compared the percentage of patients who developed antibodies for each study drug. Next, we summarized the outcomes across all studies, since the heterogeneity of the studies precluded quantitative pooling of results. Lastly, we evaluated the influence of the study design and population on the outcomes to draw conclusions about the comparability of biosimilar insulins and their reference products.

### Role of the funding source

This work was supported in part through the Johns Hopkins Center of Excellence in Regulatory Science and Innovation (U01 FD004977-03). The funding source had no role in the design and conduct of the study, analysis or interpretation of the data, and preparation or final approval of the manuscript prior to publication. Pharmerit International provided support in the form of salaries for authors EL but did not have any additional role in the study design, data collection and analysis, decision to publish, or preparation of the manuscript.

## Results

### Screening and article selection

Of the total 6945 reviewed titles and abstracts, 40 were assessed for full-text eligibility (S1 Fig). Of the 40 full-text articles, 29 were excluded as they were not RCT, not in English, and not in humans. The remaining 11 studies, all RCTs, met eligibility criteria and were included in the data synthesis [28–38]. Two open-label trials that studied LY2963016 and SAR342434 included an extension study that followed patients for up to 52 weeks to assess for immunogenicity and additional adverse events [33,35].

The 11 RCTs we included examined 5 biosimilars: LY2963016 (Eli Lilly), Basalog (Biocon), Basalin (Gan & Lee), SAR342434 (Sanofi), and MK-1293 (Merk and Co.) LY2963016, Basalog, Basalin, and MK-1293 were compared to insulin glargine (Lantus) as a reference product while SAR342434 was compared to insuin lispro (Humalog). Three trials enrolled healthy volunteers, 5 enrolled type 1 diabetics, and 2 enrolled type 2 diabetics (Table 1). One study enrolled both healthy and type 1 diabetics. We did not identify published studies for several basal and non-basal biosimilars, such as Glaritus (developed by Wockhardt and approved in India) [39], Glarvia (developed by Biocon and marketed in India) [40], Glarine (ACI Limited and used in Bangladesh) [41], Basugine (Lupin and LG Life Sciences and marketed in India) [42] and Jusline (Julphar and marketed in Middle Eastern) [43].

**Table 1:** Characteristics of trials comparing biosimilar and reference insulins.

Study, Year	Study Design	Location	Funding	BSM vs. REF	Study Length	Outcomes
***Study population*: *Healthy adults***						
**Cheng, 2010**	Single-center, randomized, single-blind, 3-period, crossover	China	NR*	Basalin vs. Lantus	24-hr per period, 2-wk washout	PK, PD, AE
**Linnebjerg, 2015**	Phase 1, 3 single-site, randomized, double-blind, 2-treatment, 4-period, crossover	Singapore, South Africa	Eli Lilly, Boehringer Ingelheim	LY IGlar vs. Lantus (REF for EU) vs. Lantus (REF for US)	24-hr per period, ≥ 1-wk washout	PK, PD, AE
**Zhang, 2017**	Phase 1, single-site, randomized, double-blind, 4-treatment, 4-period, crossover	Singapore	Eli Lilly	LY IGlar vs. Lantus	24-hr per period, ≥ 6-day washout	PK, PD, AE
**Crutchlow, 2017**	Single-dose, randomized, double-blind, single-center, crossover	NR	Merck & Co.	MK-1293 vs. Lantus (REF for EU) vs. Lantus (REF for US)	24-hr per period, ≥ 1 week washout	PK, PD, AE
***Study population*: *Type 1 diabetics***						
**Verma, 2011**	Randomized, open-label, multicenter	India	Biocon Limited	Basalog vs. Lantus	12 weeks	CE, AE, IMM
**Blevins, 2015**	Phase 3, randomized, open-label, multicenter, two-arm, active-controlled, parallel	Multinational	Eli Lilly, Boehringer Ingelheim	LY IGlar vs. Lantus	24 weeks; extended for another 28 weeks for safety studies	CE, AE, IMM
**Linnebjerg, 2016**	Randomized, double-blind, single-dose, two-period, crossover	Germany	Eli Lilly	LY IGlar vs. Lantus	42-hr per period, 1-3-wk washout	PK, PD, AE
**Kapitza, 2016**	Single-site, randomized, double-blind, single-dose, 3-period crossover	Germany	Sanofi	SAR342434 vs Humalog (REF for EU) vs Humalog (REF for US)	≥ 5-18-days washout per 2 consecutive administration	PK, PD, AE
**Garg, 2017**	Phase 3, multicenter, randomized, two-arm, parallel, open-label	Multinational	Sanofi	SAR342434 vs Humalog	26 weeks; extended for another 26 weeks for safety studies	CE, AE, IMM
**Crutchlow, 2017**	Single-dose, randomized, double-blind, single-center, crossover studies	NR	Merck & Co.	MK-1293 vs. Lantus (REF for EU)	30-hr per period, ≥1 week washout	PK, PD, AE
***Study population*: *Type 2 diabetics***						
**Rosenstock, 2015**	Phase III, randomized, multicenter, two-arm, active-controlled, double-blind, parallel	Multinational	Eli Lilly, Boehringer Ingelheim	LY IGlar vs. Lantus	24 weeks	CE, AE, IMM
**Derwahl, 2018**	Phase 3, multicenter, 6-month, randomized, open-label, two-arm parallel-group	Multinational	Sanofi	SAR342434 vs. Humalog	26-week treatment period, 1-day safety followup	CE, AE, IMM

AE adverse event, CE clinical efficacy, BSM biosimilar, REF reference biologic, IMM immunogenicity, LY IGlar = LY2963016, PK pharmacokinetics, PD pharmacodynamics, NR not reported.

*Basalin was manufactured and provided by Gan & Lee Pharmaceutical.

### Scientific quality of selected articles

Overall, the 11 trials were of moderately quality. Each of the 11 trials was judged as having a low risk of detection or attrition bias (S2 Fig). Ten of the 11 studies had an unclear risk of selection bias, primarily due to lack of information or uncertainty about the potential for bias. Similarly, 5 of the 11 trials had an unclear risk of reporting bias, while Verma (2011), Zhang (2017), and Garg (2017) had high risk and Linnebjerg (2015), Linnebjerg (2016), and Kapitza (2016) had low risk of such bias. Nine trials had low risk of performance bias, while Cheng (2010) and Derwahl (2018) had high risk. Other sources of bias that were assessed as high risk in all of the trials included the potential for conflicts of interest (e.g. authors employed by drug manufacturer or studies were funded by the drug manufacturer).

### Pharmacokinetic and pharmacodynamic outcomes

Seven trials, with sample sizes ranging from 16–171, evaluated pharmacokinetic and pharmacodynamics outcomes (S2 Table). These trials were all crossover studies [28–31, 36, 37]. Four studies included healthy adults and 3 studies evaluated patients with type 1 diabetes (S3 Table). Notably, Linnebjerg (2015) and Crutchlow (2017) compared the biosimilar LY2963016 and MK-1293, respectively, with both the US and EU version of Lantus in healthy adults. Kapitza (2016) compared SAR342434 with both the US and EU version of Humalog in type 1 diabetics.

For pharmacokinetic (PK) parameters (AUC and C_max_), one study (Linnebjerg 2016) of patients with type 1 diabetics did not have analyzable PK data. Linnebjerg (2015), Kapitza (2017), Zhang (2017), and Crutchlow (2017) all specified an equivalence margin of 80% to 125% for both AUC and C_max_ outcomes. In the Cheng trial (2010), AUC had an equivalence margin of 80% to 125% while C_max_ had an equivalence of 70% to 143%. Ratio of geometric means for each outcome for these trials was within the pre-specified margin indicating PK equivalence between the biosimilar and reference biologic.

For pharmacodynamic (PD) parameters, Linnebjerg (2015), Linnebjerg (2016) and Zhang (2017) evaluated both G_total_ and R_max_. All three studies had ratio of geometric means within the specified equivalence margin of 80% to 125%, indicating comparable PD between the two arms. In addition, Linnebjerg (2016) evaluated duration of action between the LY2963016 and Lantus groups. Results were analyzed by survival analysis with a log-rank test of equality. Time to event was defined by participants who reached duration of action after 42 hours. P value was insignificant (p value = 0.859), suggesting comparable duration of action between LY2963016 and Lantus among type 1 diabetics. This is noteworthy for long-acting insulins as patients with type 1 diabetics are regarded as the most suitable population for determining time-action profile [11]. Cheng (2010) did not include G_total_ nor did the authors specify an equivalence margin for R_max_. Crutchlow (2017) and Kapitza (2017) evaluated only R_max_ and both had ratio of geometric means within the specified equivalence margin of 80% to 125%, suggesting glucodynamic activity.

It is important to note that the Zhang (2017) study was not statistically powered to demonstrate PK or PD equivalence, but provided complementary evidence for the similarity of PK outcomes between LY IGlar and Lantus at two different doses.

### Clinical efficacy outcomes

Five of the 11 trials assessed clinical efficacy (Table 2), with 3 studies enrolling type 1 diabetics and 2 studies enrolling type 2 diabetics [32–35, 38]. These studies compared Basalog and LY2963016 to Lantus and SAR342434 to Humalog. Sample size for these studies ranged from 215–756 (S2 Table).

**Table 2:** Clinical efficacy in trials comparing biosimilar and reference insulins.

Study, Year	BSM vs REF	Analytical population and primary end point	Time point, *wk*	HbA1c endpoint, % (SD)	Change from baseline (SD)	LS Mean Difference (95% CI)
***Study population*: *Type 1 diabetics***						
**Verma, 2011**	Basalog	FAS analysis. Primary endpoint: change in HbA1c at week 12.	12	7.80 (1.24) 7.58 (1.27)	-	-
	Lantus					
**Blevins, 2015**	LY IGlar	FAS analysis. Primary endpoint: change in HbA1c at week 24.	24	7.42 (0.05)	-0.35 (0.05)	-0.108 (-0.002, 0.219)
	Lantus			7.31 (0.05)	-0.46 (0.05)	
**Garg, 2017**	SAR342434 Humalog (REF)	ITT analysis. Primary endpoint: change in HbA1c at week 26	26		-0.42 (0.05) -0.47 (0.05)	-0.06 (-0.084, 0.197)
***Study population*: *Type 2 diabetics***						
**Rosenstock, 2015**	LY IGlar	FAS analysis. Primary endpoint: change in HbA1c at week 24.	24	7.04 (0.06)	-1.29 (0.06)	-0.052 (-0.070, 0.175)
	Lantus			6.99 (0.06)	-1.34 (0.06)	
**Derwahl, 2018**	SAR342434 Humalog (REF)	ITT Population. Primary endpoint: Change in HBA1c at week 26	26	-	-0.92 (0.051) -0.85 (0.051)	-0.07 (-0.215 to 0.067)

BSM biosimilar, REF reference biologic, FAS full-analysis set, ITT Intention to treat, NR Not reported.

Baseline characteristics of patients were similar within groups for all five studies (S4 Table). The primary endpoint for the studies was change in hemoglobin A1c from baseline to the end of time point. The time point for the primary analysis differed across the trials ranging from 12 weeks to 26 weeks. All of the studies concluded non-inferiority if the mean treatment difference, including 95% CI, for treatment difference was less than or equal to a predetermined non-inferiority margin at the specified time point. All trials showed equivalence between biosimilars and reference products based on their pre-specified margins. Though not powered to test a treatment difference at 52 weeks, Blevins (2015) and Garg (2017) trials, which were extended for an additional 28 and 26 weeks, respectively, for safety assessment, also demonstrated clinical efficacy equivalence at that time point.

### Adverse events reported

All 11 trials examined adverse events within the analytical population including all patients who received at least one dose of either biosimilar or reference product (Table 3). These events included treatment emergent adverse events (TEAE), such as hypoglycemic incidences and injection site reactions, and serious adverse events (SAE) that resulted in serious injury or death.

**Table 3:** Adverse events reported in trials comparing biosimilar and reference insulins.

Study, Year	BSM vs REF	Patients, *n* (%)						Deaths, *n*	
		With ≥ 1 Adverse Event		With ≥ Serious Adverse Event		Who discontinued because of adverse events		BSM	REF
		BSM	REF	BSM	REF	BSM	REF		
**Study population: Healthy adults**									
**Cheng, 2010**	Basalin vs. Lantus	0 (0.0)	0 (0.0)	0 (0.0)	0 (0.0)	0 (0.0)	0 (0.0)	0	0
**Linnebjerg, 2015**	LY IGlar vs. Lantus (REF for EU) vs. Lantus (REF for US)	3 (-)	3 (-)	0 (0.0)	0 (0.0)	0 (0.0)	0 (0.0)	0	0
**Zhang, 2017**	LY IGlar vs. Lantus	-	-	-	-	-	-	-	-
**Crutchlow, 2017**	MK-1293 vs. Lantus (REF for EU) vs. Lantus (REF for US)	8 (7.8)	7 (3.5)	0	0	0	0	0	0
**Study population: Type 1 diabetics**									
**Verma, 2011**	Basalog vs. Lantus	61 (57)*	57 (52.8)*	1 (0.9)	1 (0.9)	2 (1.8)	0 (0.0)	0	0
**Blevins, 2015**	LY IGlar vs. Lantus	167 (62)	166 (62)	20 (8.0)	24 (9.0)	2 (1.0)	6 (2.0)	0	1
**Linnebjerg, 2016**	LY IGlar vs. Lantus	0 (0.0)	1 (5.3)	0 (0.0)	0 (0.0)	0 (0.0)	0 (0.0)	0	0
**Kapitza, 2016**	SAR342434 vs Humalog (REF for EU) vs Humalog (REF for US)	6 (20.6)	EU: 3 (10.3) US: 6 (20.6)	0 (0.0)	0 (0.0)	0 (0.0)	0 (0.0)	0	0
**Garg, 2017**	SAR342434 vs. Humalog	137 (54.5)	141 (55.5)	20 (7.9)	19 (7.5)	2 (0.8)	2 (0.8)	1	0
**Crutchlow, 2017**	MK-1293 vs. Lantus	2 (2.7)	1 (1.3)	0	0	0	0	0	0
**Study population: Type 2 diabetics**									
**Rosenstock, 2015**	LY IGlar vs. Lantus	196 (52)	184 (48)	15 (4)	18 (5)	6 (2)	11 (3)	1	1
**Derwahl, 2018**	SAR342434 vs. Humalog	118 (46.6)	108 (42.9)	14 (5.5)	27 (10.7)	7 (2.8)	6 (2.4)	1 (0.4)	2 (0.8)

For all studies, the population for AE analysis included all patients who received ≥ 1 dose of either biosimilar or reference drug.

BSM biosimilar, REF reference biologic.

Data are mean ± standard deviation, unless otherwise indicated.

*Adverse events include hypoglycemic events and other non-hypoglycemic adverse event.

The 7 trials that measured PK and PD outcomes had few to no adverse events reported for either treatment groups [28–31, 36, 37]. Participants in the Cheng (2010) trial did not experience any adverse events. Linnebjerg (2015) trial reported a total of 6 hypoglycemic events; 3 in the LY2963016 and 3 in the Lantus group. Only 1 participant in the Lantus group in Linnebjerg (2016) trial experienced a hypoglycemic event. Zhang et al (2017) did not provide numbers of AEs but reported no notable differences in the safety profiles between LY IGlar and Lantus. The most common TEAE reported in all study groups in the Kapitza trial (2017) was headache, with 5 subjects in the SAR342434 group, 4 in US-approved Humalog, and 2 in EU-approved Humalog. Likewise in Crutchlow (2017), the most common TEAE reported was injection-site pain in healthy subjects and type 1 diabetics, with 10 subjects in MK-1293 group and 8 in the reference group in healthy subjects. The low number of adverse events may be attributed to small sample sizes and short study duration.

The other 5 trials, which measured clinical efficacy and immunogenicity, included comparable proportions of patients with treatment-emergent adverse events and serious adverse events between the biosimilar and reference group. The most common adverse event across these studies was hypoglycemia. In Verma (2011), pyrexia was the most common non-hypoglycemic adverse event, with 3 events in each arm. The most common non-hypoglycemic adverse event reported in the other 4 trials was nasopharyngitis.

A total of 7 deaths occurred among these 5 studies; 3 in the biosimilar group and 4 in the reference group. Two deaths occurred in the LY2963016 group and 1 death was in the Lantus group. The reasons (hypertrophic cardiomyopathy, myocardial infarction, and lung adenocarcinoma) were not considered related to the study treatment or study design by the investigators. Additionally, two deaths occurred in the SAR342434 group due to cardiovascular events and 2 deaths in the Humalog group due to cardiopulmonary failure and bladder cancer with metastasis.

Overall, the incidences of AEs and SAEs reported for the biosimilars were similar to the reference products.

### Immunogenicity data

Five of the 11 trials [32–35, 38] assessed immunogenicity (S5 Table). Participants consisted of patients with type 1 and type 2 diabetes. Time points ranged from 12 to 52 weeks. In all five trials, immunogenicity was assessed using a validated radio immunoassay format.

Immunogenicity was examined in all patients who received one dose of either biosimilar or reference product. Two trials (Verma 2011 and Rosenstock 2015) had a higher percentage of patients developing anti-drug antibodies in the biosimilar group relative to the reference product. In the Verma trial (2011), 38.10% in the Basalog arm and 28.72% in the Lantus arm tested positive for anti-drug antibodies. In the Rosenstock trial (2015), 15% of patients developed anti-drug antibodies in the biosimilar group compared to 11% of patients in the reference product. In both cases, this was statistically not significant. Blevins (2015), Garg (2017) and Derwahl (2018) trials all had similar proportion of patients developing antibodies between the biosimilar and reference groups. Immunogenicity profiles appeared to be comparable across study groups in all studies. In two separate follow up analyses, Illag et al also supported the assessment that immunogenicity profiles were similar between LY2963016 and the respective reference product [44, 45]. An investigation by Home et al demonstrated similar immunogenicity profile between SAR342434 and its reference product [46]. Notably, the Rosenstock trial (2015) showed a lower immunogenicity for both arms compared to other trials.

### Review of trial registries for unpublished trials

S6 Table depicts the results of searches of trial registries. There were 22 studies listed in trial registries that were completed yet unpublished; 8 of these were completed in 2014 or earlier, while 5 were completed in 2015, 5 in 2016, 2 in 2017, and 2 were unknown.

## Discussion

To our knowledge, this is the first systematic review of the safety, efficacy, and immunogenicity of biosimilar insulins in comparison to their reference products. We identified 11 clinical trials comparing 2 types of biosimilar insulin glargine with its biologic originator, Lantus, and 1 type of biosimilar insulin lispro with its originator, Humalog. All of the trials we examined indicated comparable pharmacokinetic, pharmacodynamic, clinical efficacy, safety and immunogenicity outcomes.

Our results are important because of the growing commercial, regulatory and clinical importance of biosimilars in the U.S. and around the world. As more biosimilars are joining the global market, it is imperative to assess the similarity of safety and efficacy of the biosimilar to the respective originator product. While the European Medicine Agency (EMA) and US FDA have stringent regulations and assessments for comparability between biosimilar and its originator products [47–50], many other countries do not have the same regulations. Several biosimilar insulins, such as Bonglixan (Mexico), Glaritus (India), Wosulin(India) and Gensulin(India), are currently available in the marketplace, yet have not been subject to rigorous scientific scrutiny and regulatory evaluation [10, 24, 51–53]. Such data will continue to be of high interest to patients, providers and payers alike, given the inevitability of continued questions and debates about the appropriate role of biosimilar insulins and other biosimilar products in clinical practice [54, 55].

Although Basaglar, Admelog, and Lusduna were approved through a 505(b)(2) abbreviated pathway by the FDA, future biosimilar insulins are likely to be regulated through the 351(k) pathway which was designed specifically for biosimilars [56]. Under the 351(k) pathway, an approved biological product can be a biosimilar to an FDA-approved reference product or may be determined to be “interchangeable” if a higher evidentiary threshold is met [50, 57]. This distinction is important because a designation of “interchangeable” allows greater discretion on the part of dispensers to substitute a biosimilar for a reference product without a prescriber’s assent [56]. The FDA has issued a draft guidance on the required criteria that must be met to obtain such designation, such as study with a switch design [58]. Regardless of the FDA’s designation, legislation for automatic substitution rests upon each state [23]. Several states have already considered or passed legislation to allow substitution of a biosimilar for an originator product [59]. On the contrary, many countries in the EU do not allow automatic substitution [60].

Biosimilar insulins are expected to cost less than their reference products, saving the health care system as much as $44 billion through 2024 [25]. Despite these projections, their costs nevertheless remain a significant concern to patients and payers alike [61]. In contrast to generic small molecule pharmaceuticals, which on average may cost as little as 30% of their branded counterparts, cost savings from biosimilars are estimated to be far less [62], even though biologic products represent one of the costliest sectors of the pharmaceutical marketplace [63]. There are a variety of reasons that contribute to differences between the generic small molecule and biosimilar markets, including differences in the safety, manufacturing, patient and prescriber acceptance and marketing and promotion of biosimilar products [61]. While reductions in price between biosimilar and originator insulin glargine are estimated to be between 20% and 40%, it is unclear to what degree these costs will be passed on from payers to patients [25, 26].

Our review had several limitations. First, while we took a number of steps to mitigate the potential for publication bias, such as the use of a broad search strategy of multiple databases and analysis of clinical trial registries, the potential for such bias cannot be fully eliminated and our search of trial registries yielded several completed yet unpublished studies. Studies with significant and, in most cases, beneficial results are more likely to be published than those without, so our review may overstate the evidence in support of biosimilar glargine. Second, our review was limited to a small number of clinical trials, which reduces generalizability to diverse populations and routine clinical settings. Third, we did not evaluate the impact of devices used for insulin administration, such as insulin pump or pen. Biosimilar insulin pens have been shown to have higher dosage variability and different injection forces in purely technical testing, but we do not know if these differences have any effects on patient outcomes [53]. This area is becoming increasingly important to ensure accurate dosing of insulin to the patient [3, 51, 53, 64]. Finally, we did not have sufficient data to examine the interchangeability of biosimilar and originator products. None of these studies were designed as switch study in accordance to the FDA draft guidance on interchangeability between biosimilar and its reference products [58]. Furthermore, the EMA currently do not address the issue of interchangeability, but rather leaves the decision to individual states [60].

## Conclusion

Although biosimilar insulins reached the global market more than a decade ago, the first biosimilar insulin was approved in United States in 2015. As this market expands, more questions will emerge regarding the safety, effectiveness, and interchangeability of biosimilar and reference products. Relative to how commonly these products are used, little scientific evidence exists regarding these issues. However, the studies that we identified suggest similar clinical efficacy and safety of LY IGlar, MK-1293, Basalin, Basalog, and SAR342434 compared to their reference products. These biosimilars may be considered as alternative options for non-basal and basal insulin therapy in patients with type 1 and type 2 diabetes.

## Supporting information

S1 Fig Flow chart of screening process. (DOC)

S2 Fig Risk of bias in randomized controlled trials. (DOC)

S1 Table Search strategy. (DOC)

S2 Table Interventions in randomized controlled trials. (DOC)

S3 Table Pharmacokinetics and pharmacodynamics outcomes in randomized controlled trials. (DOC)

S4 Table Study populations in randomized controlled trials. (DOC)

S5 Table Immunogenicity data in randomized controlled trials. (DOC)

S6 Table List of registered trials for biosimilar insulins. (DOC)

S1 File Biosimilar insulin PRISMA checklist. (DOC)
