# Supplementary material for: Efficacy and safety of biosimilar insulins compared to their reference products: A systematic review
Source: PLoS One. 2018 Apr 18;13(4):e0195012. doi: 10.1371/journal.pone.0195012 (PMC5905882; doi:10.1371/journal.pone.0195012)
Supplement: S1 Fig — (DOC) [file pone.0195012.s001.doc]

**S1 Fig. Flow chart of screening process**

**Screening**

**Included**

**Eligibility**

**Identification**

Records identified through database searching
(n = 6945): Cochrane = 229; Embase = 3871; PubMed = 2793; LILAS = 40; South Asisan Dabase = 3; IndiaMED = 9

Additional records identified through other sources
(n=2)

Records after duplicates removed
(n = 5704): Cochrane = 225; Embase = 2694; PubMed = 2736; LILAS = 35; South Asian Database = 3; IndiaMED = 9, other sources = 2

Records screened
(n = 5704)

Records excluded
(n = 5664)

Non-insulin = 3441

Not in human = 5

Non-biosimilar = 31

Not RCT = 2173

Non-English = 14

Full-text articles assessed for eligibility
(n = 40)

Full-text articles excluded, with reasons
(n = 29)

Not RCT = 25

Not in English = 3

Not in Humans = 1

Studies included in qualitative synthesis
(n = 11)
