# Supplementary material for: Efficacy and safety of biosimilar insulins compared to their reference products: A systematic review
Source: PLoS One. 2018 Apr 18;13(4):e0195012. doi: 10.1371/journal.pone.0195012 (PMC5905882; doi:10.1371/journal.pone.0195012)
Supplement: S2 Fig — (DOC) [file pone.0195012.s002.doc]

**S2 Fig. Risk of bias in randomized controlled trials**

| **Study, Year** | **Selection Bias** | | **Performance Bias** | **Detection Bias** | **Attrition Bias** | **Reporting Bias** | **Other Bias** |
| --- | --- | --- | --- | --- | --- | --- | --- |
|  | **Random Sequence Generation** | **Allocation Concealment** | **Blinding of participants and personnel** | **Blinding of Outcome Assessment** | **Incomplete Outcome Data** | **Selective Reporting** | **Other Sources of Bias** |
| **Cheng, 2010** | ? | ? | - | + | + | ? | - |
| **Linnebjerg, 2015** | ? | ? | + | + | + | + | - |
| **Zhang, 2017** | ? | ? | + | + | + | - |  |
| **Verma, 2011** | + | + | + | + | + | - | - |
| **Blevins, 2015** | ? | ? | + | + | + | ? | - |
| **Linnebjerg, 2016** | ? | ? | + | + | + | + | - |
| **Kapitza, 2016** | ? | ? | + | + | + | + |  |
| **Garg, 2017** | ? | ? | + | + | + | - |  |
| **Rosenstock, 2015** | ? | ? | + | + | + | ? | - |
| **Crutchlow, 2017** | ? | ? | + | + | + | ? | - |
| **Derwahl, 2018** | ? | ? | - | + | + | ? | - |

Figure 2 notes:

Attrition bias based on primary endpoint identified by authors.

Other bias: risk of bias due to conflict of interest (e.g. authors employed by drug manufacturer or studies were funded by drug manufacturer)

+ low risk of bias

? unclear risk of bias (lack of information or uncertainty over the potential for bias)

- high risk of bias
