# Supplementary material for: Efficacy and safety of biosimilar insulins compared to their reference products: A systematic review
Source: PLoS One. 2018 Apr 18;13(4):e0195012. doi: 10.1371/journal.pone.0195012 (PMC5905882; doi:10.1371/journal.pone.0195012)
Supplement: S1 Table — (DOC) [file pone.0195012.s003.doc]

**S1 Table. Search Strategy**

Searched January 14, 2018

| **Database** | **Hits** | **Search terms** |
| --- | --- | --- |
| **PubMed** | 2793 | #1 "Biosimilar Pharmaceuticals"[Mesh] OR Biosimilar*[tw] OR "Follow-on biologic"[tw] OR "Follow-on biologics"[tw] OR "Follow-on biological"[tw] OR "Follow-on biologicals"[tw] OR "Subsequent entry biologic"[tw] OR "Subsequent entry biologics"[tw] OR "Subsequent entry biological"[tw] OR "Subsequent entry biologicals"[tw] OR "Similar biotherapeutic"[tw] OR "Similar biotherapeutics"[tw] OR "Similar biologicals"[tw] OR "Similar biologics"[tw] OR Biogeneric*[tw] OR "Me-too biologic"[tw] OR "Me-too biologics"[tw] OR "Me-too biological"[tw] OR "Me-too biologicals"[tw] OR "Non-innovator biologic"[tw] OR "Non-innovator biologics"[tw] OR "Non-innovator biological"[tw] OR "Non-innovator biologicals"[tw] OR “Biomimic”[tw]  #2 "glaritus"[tw] OR "glarvia"[tw] OR "basalog"[tw] OR "univia"[tw] OR "basalin"[tw] OR "bonglixan"[tw] OR "basaglar"[tw] OR "abasaglar"[tw] OR "LY2963016"[tw] OR "basagin"[tw] OR "basugine"[tw] OR "vibrenta"[tw] OR "glarine"[tw]  #3 "wosulin"[tw] OR "insugen"[tw] OR "gensulin"[tw] OR "scilin"[tw] OR "biosulin"[tw] OR "prandalin"[tw] OR "marvel"[tw] OR "solumarv"[tw] OR "isomarv"[tw] OR "combimarv"[tw] OR "solumarv"[tw] OR "combimarv"[tw] OR “julphar”[tw] OR “jusline”[tw]  #4 ("animals"[MeSH Terms]) NOT ("humans"[MeSH Terms])  #5 #1 OR #2 OR #3  #6 #5 NOT #4 |
| **EMBASE** | 3871 | #1 ("glaritus" OR "glarvia" OR "basalog" OR "univia" OR "basalin" OR "bonglixan" OR "basaglar" OR "abasaglar" OR "LY2963016" OR "basagin" OR "basugine" OR "vibrenta" OR "glarine"):ti,ab,tn  #2 ("wosulin" OR "insugen" OR "gensulin" OR "scilin" OR "biosulin" OR "prandalin" OR "marvel" OR "solumarv" OR "isomarv" OR "combimarv" OR "solumarv" OR "combimarv" OR "julphar" OR "jusline"):ti,ab,tn  #3 'biosimilar agent'/exp OR (Biosimilar* OR "Follow-on biologic" OR "Follow-on biologics" OR "Follow-on biological" OR "Follow-on biologicals" OR "Subsequent entry biologic" OR "Subsequent entry biologics" OR "Subsequent entry biological" OR "Subsequent entry biologicals" OR "Similar biotherapeutic" OR "Similar biotherapeutics" OR "Similar biologicals" OR "Similar biologics" OR Biogeneric* OR "Me-too biologic" OR "Me-too biologics" OR "Me-too biological" OR "Me-too biologicals" OR "Non-innovator" OR “Biomimic”):ti,ab  #4 #1 OR #2 OR #3  #5 ('animal'/exp) NOT ('human'/exp)  #6 #4 NOT #5 |
| **Cochrane Library** | 229 | #1 MeSH descriptor: [Biosimilar Pharmaceuticals] explode all trees  #2 Biosimilar* OR "Follow-on biologic" OR "Follow-on biologics" OR "Follow-on biological" OR "Follow-on biologicals" OR "Subsequent entry biologic" OR "Subsequent entry biologics" OR "Subsequent entry biological" OR "Subsequent entry biologicals" OR "Similar biotherapeutic" OR "Similar biotherapeutics" OR "Similar biologicals" OR "Similar biologics" OR Biogeneric* OR "Me-too biologic" OR "Me-too biologics" OR "Me-too biological" OR "Me-too biologicals" OR "Non-innovator" OR “biomimic”  #3 "glaritus" OR "glarvia" OR "basalog" OR "univia" OR "basalin" OR "bonglixan" OR "basaglar" OR "abasaglar" OR "LY2963016" OR "basagin" OR "basugine" OR "vibrenta" OR "glarine"  #4 "wosulin" OR "insugen" OR "gensulin" OR "scilin" OR "biosulin" OR "prandalin" OR "marvel" OR "solumarv" OR "isomarv" OR "combimarv" OR "solumarv" OR "combimarv" OR "julphar" OR "jusline"  #5 #1 OR #2 OR #3 OR #4 |
| **LILACS** | 40 | Biosimilar$ OR Biosimilare$ OR Biossimilare$ OR MH:D20.215.261$ OR "Follow-on biologics" OR "Follow-on biological" OR "Follow-on biologicals" OR "Subsequent entry biologic" OR "Subsequent entry biologics" OR "Subsequent entry biological" OR "Subsequent entry biologicals" OR "Similar biotherapeutic" OR "Similar biotherapeutics" OR "Similar biologicals" OR "Similar biologics" OR Biogeneric* OR "Me-too biologic" OR "Me-too biologics" OR "Me-too biological" OR "Me-too biologicals" OR "Non-innovator" OR “biomimic” OR "glaritus" OR "glarvia" OR "basalog" OR "univia" OR "basalin" OR "bonglixan" OR "basaglar" OR "abasaglar" OR "LY2963016" OR "basagin" OR "basugine" OR "vibrenta" OR "glarine" OR “wosulin” OR "insugen" OR "gensulin" OR "scilin" OR "biosulin" OR "prandalin" OR "marvel" OR "solumarv" OR "isomarv" OR "combimarv" OR "solumarv" OR "combimarv" OR "julphar" OR "jusline" |
| **ClinicalTrials.gov** | 217 | Biosimilar OR "Follow-on biologic" OR "Follow-on biologics" OR "Follow-on biological" OR "Follow-on biologicals" OR "Subsequent entry biologic" OR "Subsequent entry biologics" OR "Subsequent entry biological" OR "Subsequent entry biologicals" OR “Similar biotherapeutic” OR "Similar biotherapeutics" OR "Similar biologicals" OR "Similar biologics" OR Biogeneric OR "Me-too biologic" OR "Me-too biologics" OR "Me-too biological" OR "Me-too biologicals" OR "Non-innovator" OR “biomimic” OR "glaritus" OR "glarvia" OR "basalog" OR "univia" OR "basalin" OR "bonglixan" OR "basaglar" OR "abasaglar" OR "LY2963016" OR "basagin" OR "basugine" OR "vibrenta" OR "glarine" OR "wosulin" OR "insugen" OR "gensulin" OR "scilin" OR "biosulin" OR "prandalin" OR "marvel" OR "solumarv" OR "isomarv" OR "combimarv" OR "solumarv" OR "combimarv" OR "julphar" OR "jusline" |
| **WHO International Clinical Trials Registry Platform (ICTRP)** http://apps.who.int/trialsearch/ | 1470 | "glaritus" OR "glarvia" OR "basalog" OR "univia" OR "basalin" OR "bonglixan" OR "basaglar" OR "abasaglar" OR "LY2963016" OR "basagin" OR "basugine" OR "vibrenta" OR "glarine" OR “wosulin" OR "insugen" OR "gensulin" OR "scilin" OR "biosulin" OR "prandalin" OR "marvel" OR "solumarv" OR "isomarv" OR "combimarv" OR "solumarv" OR "combimarv" OR "julphar" OR "jusline" OR Biosimilar OR "Similar biologicals" OR "Follow-on biologic" OR "Follow-on biologics" OR "Follow-on biological" OR "biosimilar agent" OR "Follow-on biologicals" OR "Subsequent entry biologic" OR "Subsequent entry biologics" OR "Subsequent entry biological" OR "Subsequent entry biologicals" OR "Similar biotherapeutic" OR "Similar biotherapeutics" OR "Similar biologics" OR Biogeneric* OR "Me-too biologic" OR "Me-too biologics" OR "Me-too biological" OR "Me-too biologicals" OR "Non-innovator" OR “biomimic” |
| **EU Clinical Trials Register** www.clinicaltrialsregister.eu Searched on August 18, 2016 | 63 | "glaritus" OR "glarvia" OR "basalog" OR "univia" OR "basalin" OR "bonglixan" OR "basaglar" OR "abasaglar" OR "LY2963016" OR "basagin" OR "basugine" OR "vibrenta" OR "glarine" OR “wosulin” OR "insugen" OR "gensulin" OR "scilin" OR "biosulin" OR "prandalin" OR "marvel " OR "solumarv" OR "isomarv" OR "combimarv" OR "solumarv" OR "combimarv" OR "julphar" OR "jusline" OR Biosimilar OR "Similar biologicals" OR "Follow-on biologic" OR "Follow-on biologics" OR "Follow-on biological" OR "biosimilar agent" OR "Follow-on biologicals" OR "Subsequent entry biologic" OR "Subsequent entry biologics" OR "Subsequent entry biological" OR "Subsequent entry biologicals" OR "Similar biotherapeutic" OR "Similar biotherapeutics" OR "Similar biologics" OR Biogeneric* OR "Me-too biologic" OR "Me-too biologics" OR "Me-too biological" OR "Me-too biologicals" OR "Non-innovator" OR “biomimic” |
| **South Asian Database of Controlled Clinical Trials  (SADCCT)** http://cochrane-sadcct.org/ | 3 | "glaritus" OR "glarvia" OR "basalog" OR "univia" OR "basalin" OR "bonglixan" OR "basaglar" OR "abasaglar" OR "LY2963016" OR "basagin" OR "basugine" OR "vibrenta" OR "glarine" OR “wosulin” OR "insugen" OR "gensulin" OR "scilin" OR "biosulin" OR "prandalin" OR "marvel " OR "solumarv" OR "isomarv" OR "combimarv" OR "solumarv" OR "combimarv" OR "julphar" OR "jusline" OR Biosimilar OR Biologic |
| **IndiaMED** http://indmed.nic.in/ | 9 | "glaritus" OR "glarvia" OR "basalog" OR "univia" OR "basalin" OR "bonglixan" OR "basaglar" OR "abasaglar" OR "LY2963016" OR "basagin" OR "basugine" OR "vibrenta" OR "glarine" OR “wosulin” OR "insugen" OR "gensulin" OR "scilin" OR "biosulin" OR "prandalin" OR "marvel " OR "solumarv" OR "isomarv" OR "combimarv" OR "solumarv" OR "combimarv" OR "julphar" OR "jusline" OR Biosimilar |
