# Supplementary material for: Efficacy and safety of biosimilar insulins compared to their reference products: A systematic review
Source: PLoS One. 2018 Apr 18;13(4):e0195012. doi: 10.1371/journal.pone.0195012 (PMC5905882; doi:10.1371/journal.pone.0195012)
Supplement: S2 Table — (DOC) [file pone.0195012.s004.doc]

**S2 Table. Interventions in randomized controlled trials**

| **Study, Year** | **BSM vs REF** | **Completed/Enrolled, *n/N*** | **Dose** | **Frequency** |
| --- | --- | --- | --- | --- |
|  |  |  |  |  |
| ***Study population: Healthy adults*** | | | | |
| **Cheng, 2010** | Basalin vs. Lantus | 16/16 | 0.4 units/kg | Single dose administration per period |
| **Linnebjerg, 2015** | LY IGlar vs. Lantus (REF for EU)  LY IGlar vs. Lantus (REF for US) | 82/91  78/80 | 0.5 units/kg  0.5 units/kg | Single dose administration per period |
| **Zhang, 2017** | LY IGlar vs. Lantus | 23/24 | 0.3 units/kg  0.6 units/kg | Single dose administration per period |
| **Crutchlow, 2017** | MK-1293 vs. Lantus | 70/76 | 0.4 units/kg | Single dose administration per period |
| ***Study population: Type 1 diabetics*** | | | | |
| **Verma, 2011** | Basalog vs. Lantus | 178/215 | Same dose as pre-study basal insulin | Once daily |
| **Blevins, 2015** | LY IGlar vs. Lantus | At 24 weeks: 508/535  At 52 weeks: 492/535 | Same dose as pre-study basal insulin | Once daily |
| **Linnebjerg, 2016** | LY IGlar vs. Lantus | 20/20 | 0.3 units/kg | Single dose administration per period |
| **Kapitza, 2016** | SAR342434 vs Humalog (REF for EU) vs Humalog (REF for US) | 28/30 | 0.3 units/kg | Single dose administration per period |
| **Garg, 2017** | SAR342434 vs Humalog | At 26 weeks: 480/507  At 52 weeks: 461/507 | Unit-to-unit conversion from pre-study insulin lispro or insulin aspart dose; adjustment; adjust as needed to achieve target plasma glucose goal | Pre-prandial and post-prandial as needed if allowed |
| **Crutchlow, 2017** | MK-1293 vs. Lantus | 70/76 | 0.4 units/kg | Single dose administration per period |
| ***Study population: Type 2 diabetics*** | | | | |
| **Rosenstock, 2015** | LY IGlar vs. Lantus | 666/756 | Same dose as pre-study Lantus dose  Insulin-naïve patients = 10 units | Once daily |
| **Derwahl, 2018** | SAR342434 vs. Humalog | 458/505 | Unit-to-unit conversion from pre-study insulin lispro or insulin aspart dose; adjustment; adjust as needed to achieve target plasma glucose goal dose | Pre-prandial and post-prandial as needed if allowed |

*excluding Lantus (EU) vs. Lantus (US) group
