# Supplementary material for: Efficacy and safety of biosimilar insulins compared to their reference products: A systematic review
Source: PLoS One. 2018 Apr 18;13(4):e0195012. doi: 10.1371/journal.pone.0195012 (PMC5905882; doi:10.1371/journal.pone.0195012)
Supplement: S3 Table — (DOC) [file pone.0195012.s005.doc]

**S3 Table. Pharmacokinetics and pharmacodynamics outcomes in randomized controlled trials**

| **Study, Year** | **BSM vs REF** | **Definition of analysis population** | **BSM, N** | | **REF, N** | **Outcomes** | **Geometric Means Ratio (%) BSM/REF (90% CI)** |
| --- | --- | --- | --- | --- | --- | --- | --- |
| ***Study population: Healthy adults*** | | | | | | |  |
| **Cheng, 2010** | Basalin vs. Lantus | All participants (no randomized participants discontinued the study) | - | - | | AUC [0-24]  Cmax  Rmax | - |
| **Linnebjerg, 2015** | LY IGlar vs. Lantus (REF for EU) | All participants who received 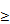1 dose of study drug | 79  80  80  80 | 80  80  80  80 | | AUC [0-24h]  Cmax  Gtotal  Rmax | 91.0 (87.0 – 96.0)  95.0 (90.0 – 100.0)  95.0 (91.0 – 100.0)  99.0 (94.0 – 104.0) |
|  | LY IGlar vs. Lantus (REF for US) | All participants who received 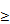1 dose of study drug | 87  88  88  88 | 89  89  88  88 | | AUC [0-24h]  Cmax  Gtotal  Rmax | 90.0 (86.0 – 94.0)  92.0 (87.0 – 96.0)  91.0 (85.0 – 98.0)  93.0 (88.0 – 98.0) |
| **Zhang, 2017** | LY IGlar vs. Lantus | All participants who received 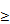1 dose of study drug    At 0.3 units/kg dose      At 0.6 units/kg dose |  | | |  |  |
|  |  |  | 23  23  23  23 | 23  23  23  23 | | AUC [0-24h]  Cmax  Gtotal  Rmax | 103.0 (91.0 – 116.0)  103.0 (92.0 – 115.0)  98.0 (78.0 – 124.0)  104.0 (87.0 – 125.0) |
|  |  |  | 24  24  24  24 | 24  24  24  24 | | AUC [0-24h]  Cmax  Gtotal  Rmax | 107.0 (95.0 – 121.0)  103.0 (92.0 – 116.0)  87.0 (70.0 – 109.0)  94.0 (79.0 – 112.0) |
| **Crutchlow, 2017** | MK-1293 vs. Lantus (REF for EU) | All participants who received 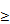1 dose of study drug | 102 102  102 | 100 100  100 | | AUC [0-24h] Cmax  Rmax | 97.0 (92 – 102)  100.0 (95 – 105)  91.0 (84.0 – 99.0) |
|  | MK-1293 vs. Lantus (REF for US) | All participants who received 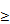1 dose of study drug | 102 102  102 | 100  100  100 | | AUC [0-24h] Cmax  Rmax | 98.0 (93 – 103)  101.0 (96 – 107)  96.0 ( 87 – 106) |
| ***Study population: Type 1 diabetics*** | | | | | | | |
| **Linnebjerg, 2016** | LY IGlar vs. Lantus | All participants who received 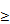1 dose of study drug  Participants who reached duration of action after 42 hours | 20  20  13 | 19  19  13 | | Gtotal  Rmax  Duration of action | 77.0 (46.0 – 130.0)  91.0 (52.0 – 161.0)  * |
| **Kapitza, 2016** | SAR342434 vs Humalog (REF for EU) | All participants who received 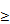1 dose of study drug | -  -  - | -  -  - | | AUC  Cmax  Rmax | 97.0 (94.0 – 100.0)  96.0 (89.0 – 104.0)  107.0 (99.0 – 114.0) |
|  | SAR342434 vs Humalog (REF for US) | All participants who received 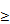1 dose of study drug | -  -  - | -  -  - | | AUC  Cmax  Rmax | 95.0 (92.0 – 99.0)  97.0 (89.0 – 105.0)  104.0 (98.0 – 110.0) |
| **Crutchlow 2017** | MK-1293 vs. Lantus | All participants who received 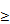1 dose of study drug | 74 74  74 | 75 75  74 | | AUC [0-24h] Cmax  Rmax | 97.0 (91 – 102)  97.0 (93 – 103)  96.0 (91.0 – 102)** |

BSM biosimilar, REF reference biologic

Units: AUC (pmol*h/L), Cmax (pmol/L), Gtotal (mg/kg), Rmax (mg/kg/min)

*survival analysis was performed with a log-rank test of equality

**95% Confidence Interval
