# Supplementary material for: Efficacy and safety of biosimilar insulins compared to their reference products: A systematic review
Source: PLoS One. 2018 Apr 18;13(4):e0195012. doi: 10.1371/journal.pone.0195012 (PMC5905882; doi:10.1371/journal.pone.0195012)
Supplement: S4 Table — (DOC) [file pone.0195012.s006.doc]

**S4 Table. Study populations in randomized controlled trials**

| **Study, Year** | **Drug Group (Sample Size)** | **Age, years** | **Male (%)** |  | **Race/Ethnicity, %** | | | |  | **Clinical Baseline Values** | | | |
| --- | --- | --- | --- | --- | --- | --- | --- | --- | --- | --- | --- | --- | --- |
|  |  |  |  |  | **White** | **African American** | **Asian** | **Other** |  | **BMI, kg/m^2^** | **Body Wt, kg** | **HbA1c, (%)** | **FPG, mg/dl** |
| ***Study population: Healthy adults*** | |  |  |  |  |  |  |  |  |  |  |  |  |
| **Cheng, 2010*** | Basalin vs Lantus (total *n*=16) | 24.4 (3.8) | 100 |  | - | - | - | - |  | 21.06 (1.7) | - | - | - |
| **Linnebjerg, 2015** | LY IGlar vs. Lantus (REF for EU)  (total *n*=80)  LY IGlar vs. Lantus (REF for US)  (total *n*=91) | 32 (10.9)  32.7 (9) | 70  93.4 |  | 80  1.1 | 12.5  98.9 | 0.0  0.0 | 7.5  0.0 |  | 24.9 (3.2)  24.1 (2.8) | 74.8 (12.5)  70.2 (10.3) | -  - | -  - |
| **Zhang, 2017** | LY IGlar vs. Lantus (total *n*=24) | 32.1 (7.7) | 83.8 |  | 0 | 0 | 100 | 0 |  | 22.7 (2.8) | 66.6 (9.2) | - | - |
| **Crutchlow, 2017** | MK-1293 vs. Lantus (REF for EU) vs. Lantus (REF for US) (total *n* = 96) | 28.1 (6.5) | 100 |  | 72.5 | 19.3 | 4.6 | 3.6 |  | 25.2 (2.4) | - | - | - |
| ***Study population: Type 1 diabetics*** | |  |  |  |  |  |  |  |  |  |  |  |  |
| **Verma, 2011** | Basalog (*n*=107)  Lantus (*n*=108) | 31.45 (11.8)  28.32 (10.3) | 53.3  56.1 |  | 0.0 | 0.0 | 100* | 0.0 |  | 21.96 (2.8)  21.61 (3.1) | 56.87 (9.9)  56.62 (10.3) | 7.99 (1.2)  7.83 (1.3) | 145.0 (63.9)  146.5 (70.3) |
| **Blevins, 2015** | LY IGlar (*n*=268)  Lantus (*n*=267) | 41 (14)  41 (13) | 58  58 |  | 74  75 | 3  1 | 18  19 | 5  5 |  | 26 (4)  25 (4) | 76 (17)  75 (15) | 7.75 (1.1)  7.79 (1.0) | 151 (54)  147 (54) |
| **Linnebjerg, 2016** | LY IGlar vs. Lantus (total *n*=20) | 41.5 (9.1) | 100 |  | 100 | 0.0 | 0.0 | 0.0 |  | 25.6 (2.4) | 84.1 (9.8) | 7.99 (0.6) | - |
| **Kapitza, 2016** | SAR342434 vs Humalog (REF for EU) vs Humalog (REF for US) (total *n*=30) | 44.0 (9.4) | 100 |  | 100 | 0 | 0 | 0 |  | 24.9 (2.1) | 78.3 (8.1) | 7.6 (0.7) | - |
| **Garg, 2017** | SAR342434 (*n*=253)  Humalog (*n*=254) | - | - |  | - | - | - | - |  | - | 77.7 (14.8)  76.7 (16.8) | 8.08 (0.8)  8.00 (0.6) | 182.5 (79.3)  181.1 (73.7) |
| **Crutchlow, 2017** | MK-1293 vs. Lantus (total *n* = 70) | 33.4 (11.7) | 63.2 |  | 92.1 | 2.6 | 2.6 | 2.7 |  | 23.3 (1.4) | - | - | - |
| ***Study population: Type 2 diabetics*** | |  |  |  |  |  |  |  |  |  |  |  |  |
| **Rosenstock, 2015** | LY IGlar (*n*=376)  Lantus (*n*=380) | 59 (10)  59 (10) | 48  52 |  | 80  77 | 7  8 | 8  9 | 5  6 |  | 32 (6)  32 (5) | 90 (20)  90 (19) | 8.34 (1.1)  8.31 (1.1) | 159 (45)  160 (44) |
| **Derwahl, 2018** | SAR342434 (*n* = 228)  Humalog (*n* = 230) | 62.1 (9.4)  62.8 (8.9) | 53.8  52.4 |  | 90.1  86.5 | 5.5  6.7 | 4.3  6.3 | 0  0.4 |  | 32.3 (4.8)  32.1 (4.8) | 92.2 (17.5)  91.2 (17.) | 8.00 (0.9)  8.03 (0.9) | 150.5 (48.1)  147.4 (50.5) |

*Demographic not reported, but study is conducted in China and India, respectively.

Data are mean
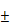
 standard deviation, unless otherwise indicated.
