# Supplementary material for: Efficacy and safety of biosimilar insulins compared to their reference products: A systematic review
Source: PLoS One. 2018 Apr 18;13(4):e0195012. doi: 10.1371/journal.pone.0195012 (PMC5905882; doi:10.1371/journal.pone.0195012)
Supplement: S5 Table — (DOC) [file pone.0195012.s007.doc]

**S5 Table. Immunogenicity data in randomized controlled trials**

| **Study, Year** | **BSM vs REF** | **Time Point, *Wk*** | **BSM, %** | **REF, %** |
| --- | --- | --- | --- | --- |
| **Verma, 2011** | Basalog vs. Lantus | 12 | 38.10 | 28.72 |
| **Blevins, 2015** | LY IGlar vs. Lantus | 24  52 | 30  40 | 34  39 |
| **Garg, 2017** | SAR342434 vs Humalog | 52 | 28.5 | 27.3 |
| **Rosenstock, 2015** | LY IGlar vs. Lantus | 24 | 15 | 11 |
| **Derwahl, 2018** | SAR342434 vs. Humalog | 26 | 30.8 | 29.2 |

BSM biosimilar, REF reference biologic
