# Supplementary material for: Efficacy and safety of biosimilar insulins compared to their reference products: A systematic review
Source: PLoS One. 2018 Apr 18;13(4):e0195012. doi: 10.1371/journal.pone.0195012 (PMC5905882; doi:10.1371/journal.pone.0195012)
Supplement: S6 Table — (DOC) [file pone.0195012.s008.doc]

**S6 Table. List of registered trials for biosimilar insulins**

| **Trial ID (Primary Sponsor)** | **Study Design** | **Study Population** | **BSM vs. REF (Status)** |
| --- | --- | --- | --- |
| **COMPLETED: With full text publications** | | | |
| NCT00532766 (King Saud University) | Double blind, crossover | Healthy adults | Jusline vs. REF Humulin Regular, NPH, and Premixed Regular/NPH (30/70) (Completed: Feb 2007) |
| NCT01421459 (Eli Lilly and Company) | Phase 3, double blind, parallel | Type 2 diabetics | LY2963016 vs. REF Lantus® (Completed: Sept. 2012) |
| NCT01421147 (Eli Lilly and Company) | Phase 3, open label, parallel | Type 1 diabetics | LY2963016 vs. REF Lantus® (Completed: April 2013) |
| NCT01600950 (Eli Lilly and Company) | Phase 1 double blind, crossover | Type 1 diabetics | LY2963016 vs. REF Lantus® (Completed: July 2012) |
| NCT01476345 (Eli Lilly and Company) | Phase 1, double blind, cross over | Healthy adults | LY2963016 vs. REF Lantus® (Completed: July 2012) |
| NCT01688635 (Eli Lilly and Company) | Phase 1, double blind, crossover | Health adults | LY2963016 vs. REF Lantus® (Completed: Feb. 2013) |
| NCT02294474 (Sanofi) | Phase 3, open-label, randomized | Type 2 diabetics | SAR342434 vs REF Humalog ® (Completed: Feb. 2016) |
| NCT02273180 (Sanofi) | Phase 3, open-label, randomized | Type 1 diabetics | SAR342434 vs REF Humalog ® (Completed: July 2016) |
| NCT02059174 (Merck Sharp & Dohme Corp.) | Double-blind, randomized, crossover | Healthy adults and Type 1 diabetics | MK-1293 vs REF Lantus ® (April 2015) |
| NCT02273258 (Sanofi) | Double-blind, randomized, crossover | Type 1 diabetics | SAR342434 vs REF Humalog ® (Completed: July 2013) |
| NCT01634165 (Eli Lilly and Company) | Phase 1, double-blind, crossover | Healthy adults | LY2963016 vs. REF Lantus® (Completed: Sept. 2012) |
| CTRI/  2008/091/000226 (Biocon) | Open-label | Type 1 diabetics | Basalog vs. REF Lantus® (Completed: Dec 2009) |
| **COMPLETED: No full text publication available** | | | |
| NCT02634515 (Julphar Gulf Pharmaceutical Industries) | Phase 1 double-blind, crossover | Healthy adults | Julphar Insulin R vs. REF  Huminsulin® Normal (Completed: Feb. 2015) |
| NCT02631928 (Julphar Gulf Pharmaceutical Industries) | Phase 1 double-blind, crossover | Healthy adults | Julphar Insulin 30/70 vs. REF Huminsulin® Profil III (Completed: June 2016) |
| NCT02227862 (Mylan Inc.) | Phase 3 open-label, parallel | Type 1 diabetics | Mylan's insulin glargine vs. REF Lantus® (Completed: July 2016) |
| NCT02227875 (Mylan Inc.) | Phase 3 open-label, parallel | Type 2 diabetics | Mylan's insulin glargine vs. REF Lantus® (Completed: Dec. 2015) |
| NCT02955953 (Eli Lilly and Company) | Phase 1, double blind, crossover | Healthy adults | LY2963016 U200 vs. LY2963016 U100 (Completed: Jan. 2017) * |
| NCT01374178 (Eli Lilly and Company) | Phase 1, open label cross over | Healthy adults | LY2963016 vs. REF Lantus® (Completed: July 2011) |
| NCT02302716 (Eli Lilly and Company) | Phase 3, open label, parallel | Type 2 diabetics | LY2963016 vs. REF Lantus® (Completed: July 2016) |
| NCT02059187 (Merck Sharp & Dohme Corp.) | Phase 3 open label, parallel | Type 2 diabetics | MK-1293 vs. REF Lantus® (Completed: March 2015) |
| NCT02059161 (Merck Sharp & Dohme Corp.) | Phase 3 open label, parallel | Type 1 diabetics | MK-1293 vs. REF-Lantus® (Completed: Nov. 2015) |
| NCT00719108 (Wockhardt) | Phase 1 double blind, crossover | Healthy adults | Wosulin R vs. REF Actrapid  (Completed: Sept. 2008) |
| NCT00772265 (Wockhardt) | Phase 1 double blind, crossover | Healthy adults | Wosulin N vs. REF Novolin N (Completed: April 2011) |
| NCT00752180 (Wockhardt) | Phase 1 double blind, crossover | Type 1 diabetic | Wosulin R vs. REF Actrapid (Completed: March 2009) |
| NCT01358435 (Wockhardt) | Phase 1 double blind, crossover | Healthy adults | Wosulin 70/30 vs. REF Novolin® 70/30 (Completed: May 2011) |
| NCT00596063 (Wockhardt) | Phase 1 double blind, crossover | Healthy adults | Wosulin R vs. REF Novolin R (Completed: Feb. 2008) |
| NCT01357603 (Wockhardt) | Phase 1 double blind, crossover | Type 1 diabetics | Glaritus™ vs. REF Lantus (Completed: Aug 2012) |
| NCT02506647 (Gan and Lee Pharmaceuticals) | Phase 1 double blind, 2-way crossover | Type 1 diabetics | Gan & Lee insulin glargine vs. REF Lantus (Completed: Sept 2016) |
| EudraCT No.: 2014-000747-32 (Mylan GmbH) | Phase 3, open label, parallel | Type 1 diabetics | Mylan's Insulin Glargine vs. REF-Lantus® (Completed July 2016) |
| EudraCT No.: 2014-000881-23  (Mylan GmbH) | Phase 3 open label, parallel | Type 2 diabetics | Mylan's Insulin Glargine vs. REF-Lantus® (Completed: Dec. 2015) |
| EudraCT No.: 2010-018354-12  (Biocon S.A.) | Phase 3 open label, parallel | Type 1 diabetics | Insugen R and Insugen N vs. REF Actrapid and Insulatard (Completed: July 2012) |
| EudraCT No.: 2015-004353-40 (Mylan GmbH) | Phase 3 open label, parallel | Type 1 diabetics | Mylan's Insulin Glargine vs. REF Lantus® (Completed: Mar. 2017) |
| CTRI/2015/06/005890  (Wockhardt) | Double blind, 4-period crossover | Healthy adults | Glaritus™ vs. REF Lantus (Completed date: ?) |
| CTRI/2016/05/006959 (BioGenomics) | Phase 1 double blind, 2-period crossover | Healthy adults | BioGenomics Insulin Aspart  vs. REF NovoRapid (Completed:?) |
| **ON-GOING** | | | |
| NCT01352663 (Wockhardt) | Phase 3 open label, parallel | Type 1 diabetics | Glaritus vs. REF Lantus (Estimated completion date: Jan. 2017) ** |
| CTRI/2011/11/002173 (Wockhardt) | Phase 4 open label, parallel | Type 1 diabetics | Glaritus™ vs. REF Lantus (Estimated completion date: ?) |
| CTRI/2015/05/005808  (Wockhardt) | Phase 4 prospective, open label, parallel | Type 2 diabetics | Glaritus™ vs. REF Lantus (Estimated completion date: ?) |
| NCT02634528 (Julphar Gulf Pharmaceutical Industries) | Phase 1 double-blind, crossover | Healthy adults | Julphar Insulin N vs. REF Huminsulin® Basal  (Estimated completion date: Oct. 2017) |
| CTRI/2016/07/007117 (Wockhardt) | Phase 1 double blind, 2-period crossover | Healthy adults | Wosulin R (200 IU) vs. REF Wosulin R (100 IU)  (Estimated completion date: ?) |
| EudraCT No.: 2017-001450-34 (Gan & Lee) | Phase 3, open label, randomized | Type 1 diabetics | Gan & Lee insulin glargine vs REF Lantus® (Estimated completion date: ?) |
| EudraCT No.: 2016-004691-22 (Rechon) | Phase 3, open label, parallel | Type 1 diabetics | Rechon Insulin vs. REF Humulin R (Estimated completion date: ?) |

BSM biosimilar, REF reference biologic *This study was not compared to a reference product **The recruitment status of this study is unknown
